# The efficacy of sensory nerve coaptation in DIEP flap breast reconstruction – Preliminary results of a double-blind randomized controlled trial

**DOI:** 10.1016/j.breast.2024.103691

**Published:** 2024-02-09

**Authors:** Jeske M. Bubberman, Lloyd Brandts, Sander M.J. van Kuijk, René R.W.J. van der Hulst, Stefania M.H. Tuinder

**Affiliations:** a Department of Plastic, Reconstructive and Hand Surgery, Maastricht University Medical Center, Maastricht, the Netherlands; b GROW – Research Institute for Oncology and Reproduction, Maastricht University, Maastricht, the Netherlands; c Department of Clinical Epidemiology and Medical Technology Assessment, Maastricht University Medical Center, Maastricht, the Netherlands

**Keywords:** Breast cancer, Autologous breast reconstruction, DIEP flap, Sensibility, Sensation

## Abstract

**Background:**

Sensory nerve coaptation has great potential to restore sensation after autologous breast reconstruction. However, blinded and randomized studies are lacking. We therefore present the preliminary results of our ongoing double-blinded randomized controlled trial that compares sensory recovery of innervated versus non-innervated DIEP flaps.

**Methods:**

Patients who underwent DIEP flap breast reconstruction between July 2019 and February 2022 were included and randomized. The anterior cutaneous branch of the second or third intercostal nerve was coapted. Pre- and postoperative sensory testing was performed with Semmes-Weinstein Monofilaments, Pressure Specified Sensory Device, and a thermostimulator, for tactile and temperature thresholds.

**Results:**

This interim analysis comprised 41 patients contributing 29 innervated and 38 non-innervated breasts. At 24 months of follow-up, the mean monofilament value of the flap skin was lower in innervated than in non-innervated flaps (4.48 vs. 5.20, p = 0.003). Touch thresholds were lower the center of the innervated flaps (47.8 vs. 71.2 g/mm^2^, p = 0.036), and heat pain was more often imperceptible in non-innervated flaps (42.1% vs. 10.3%, p = 0.004). No adverse events were associated with sensory nerve coaptation.

**Conclusions:**

These preliminary results indicate superior sensibility and recovery of protective sensation in innervated compared with non-innervated DIEP flaps. Although the results of the completed trial must be awaited to establish the full clinical impact, including highly anticipated quality of life outcomes, we encourage continuation of scientific and clinical efforts in this promising technique.

## Highlights

• Sensibility is an important outcome after autologous breast reconstruction.
• DIEP flaps can be innervated by coaptation of intercostal nerve branches.
• Innervated DIEP flaps have improved postoperative sensibility.
• Protective sensation more often returns in innervated DIEP flaps.
• Nerve coaptation is a meaningful and relevant addition to breast reconstruction.

## Conflicts of interest and source of funding

None of the authors has a financial interest in any content of this article. This research received funding from the Dutch Cancer Society and 10.13039/100016244Health Foundation Limburg (Cancer Research Fund). No funding was received from commercial agencies.

## 1 Introduction

Lack of sensation after breast reconstruction negatively affects patient satisfaction [1]. While a variety of autologous options yielding aesthetically favorable results are available, functional outcomes are still suboptimal as sensation recovers poorly [2,3]. This causes dissatisfaction and potential harm [4,5]. Sensation used to be an undervalued outcome after breast reconstruction, but now attracts substantial public and scientific attention.

Sensory nerve coaptation is an acknowledged technique to innervate flaps for autologous breast reconstruction. Recent studies indicate superior sensory recovery in innervated flaps [[6], [7], [8], [9], [10]]. Subsequently, better sensibility improves quality of life [11,12]. Therefore, sensory nerve coaptation is increasingly recognized as a valuable addition to autologous breast reconstruction.

However, innervation of flaps is still not widely adopted in clinical practice. Previous research is attenuated by inconsistencies in methodology and sensory assessment, and double-blind randomized studies are lacking. Therefore, current evidence is still insufficient to impact clinical practice. Double-blind randomized studies are a crucial next step.

We initiated the first double-blind randomized controlled trial (RCT), comparing sensory recovery of innervated and non-innervated deep inferior epigastric artery perforator (DIEP) flaps. Here, we present the results of the interim analysis of the first cohort that completed the follow-up. We provide these preliminary data as an update of current knowledge and an answer to the rising curiosity among plastic surgeons worldwide.

## 2 Methods

We present the preliminary results of a subgroup of our prospective, double-blind RCT (Dutch Trial Register: NL7291), reported in accordance with the CONSORT statement. This single-center, multi-surgeon study is ongoing at the time of writing at Maastricht University Medical Center in the Netherlands. The ethical committee and institutional review board gave approval (METC 18–035/NL67335.068.18). The study was conducted in accordance with the ethical standards of the Declaration of Helsinki. The study started in June 2019 and is estimated to finish in 2024. Sample size was calculated on the outcome quality of life, measured by the BREAST-Q. This interim analysis comprises the results of the patients that completed 24 months follow-up by June 2022. This time point was chosen pragmatically, three years after initiation of the RCT; there was no selection of the participants included in this interim analysis, the only inclusion criterium was having completed 24 months follow-up.

### 2.1 Patients

The patients were included at our outpatient clinic. Inclusion criteria were: female sex, age ≥18 years, and undergoing post-mastectomy DIEP-flap breast reconstruction. Exclusion criteria were: comorbidities affecting sensibility, active smoking, and BMI ≥35 kg/m^2^. All patients gave written informed consent, and were randomly assigned to receive either innervated or non-innervated DIEP-flap breast reconstruction. Block-randomization was applied by an independent third party (Clinical Trial Center Maastricht). The patients were assigned to either group via the sealed envelope method. Patients and researchers were blinded to the allocation. Patient characteristics were collected through chart review. Surgical details and complications were documented.

### 2.2 Surgical procedure

All patients underwent an immediate or delayed mastectomy. Immediate reconstructions were always after skin-sparing mastectomies, with a periareolar or lollipop incision. Delayed reconstructions were after prior conventional mastectomies, or prior implant reconstructions (after skin-sparing mastectomies). Nipple-sparing mastectomies were not excluded, but are not represented in this interim analysis since these were rarely performed in our center during the inclusion period.

Non-innervated DIEP-flap breast reconstruction was performed according to current standard practice [13]. Additionally, in innervated DIEP-flap breast reconstructions, a sensory nerve coaptation was performed as described by Spiegel et al. [14] At the abdomen, a sensory nerve was carefully dissected together with the vascular pedicle. The anterior cutaneous branch of the second or third intercostal nerve was dissected as recipient nerve. After patency of the vascular anastomoses was ensured, the nerve was coapted by direct end-to-end coaptation using 9-0 nylon epineural sutures and fibrin sealant.

### 2.3 Outcomes

The outcomes are sensory recovery and quality of life. This interim analysis focuses on the sensory outcomes and does not include quality of life outcomes, which remain reserved for the analysis of the completed trial to ensure statistical power. The primary outcome of this interim analysis is tactile sensibility measured with Semmes-Weinstein Monofilaments.

Sensory recovery was assessed preoperatively, and at 3, 6, 12, 18 and 24 months postoperatively; and comprised tactile and thermal threshold testing.

Tactile thresholds were assessed with Semmes-Weinstein monofilaments (SWM) using a 20-piece SWM kit [15]. The index numbers (1.65–6.65) represent the logarithm of the force in tenths of milligrams required to bend the monofilament. Nine areas were assessed in random sequence: each quadrant of the breast, each quadrant of the areola, and the nipple (Fig. A1). After reconstruction, areas 5–9 are always located on the skin paddle of the flap, regardless of reconstruction timing. In large skin paddles, areas 2 and 3 are located on the flap skin as well. Mean sensibility in native and flap skin were determined per case, thereby accounting for individual differences in flap size or shape. According to the manufacturer's instructions, SWM values are categorized into normal sensation, diminished light touch, diminished protective sensation, loss of protective sensation, and deep pressure sensation only.

Tactile thresholds were secondly assessed with the Pressure Specified Sensory Device® (PSSD; range 1–100 g/mm^2^) [16]. Thresholds for one-point static and one-point dynamic touch were tested in three areas: center of the flap, upper medial quadrant, and lower lateral quadrant. The means of three attempts were documented per area. When static and dynamic touch thresholds were not perceived at 100 g/mm^2^, this was considered loss of protective sensation.

Thermal thresholds were assessed using PATHWAY Model ATS (Medoc, Israel) with a 30 × 30mm probe that heats and cools in a preset pattern [17]. Warmth detection, heat pain, cold detection and cold pain thresholds were assessed using the method of limits, in the same three areas as the PSSD. The means of three attempts were documented. When a thermal threshold was not perceived within the range of 0–50 °C, this was considered loss of protective sensation.

Since inter-rater variation is a known source of suboptimal reliability in sensory evaluation, variation in examiners was limited [18]. Patients were evaluated by either the research nurse or the clinical researcher who received the same training and instructions, to diminish variability in protocol execution.

### 2.4 Statistical analysis

The data was presented and analyzed according to the “as treated” principle, as the aim is to investigate the efficacy and feasibility of a sensory nerve coaptation. Baseline characteristics are presented as mean ± standard deviation (SD), median [25th – 75th percentile], or absolute count (proportion), as appropriate.

For analysis of sensory recovery, the breast areas were analyzed separately and additionally, the means for flap skin, native skin, and total breast skin were determined. When a stimulus was not perceived, this was substituted by the upper or lower limit of the measurable range. Continuous variables were compared using a multilevel linear regression model, in which correlated measurements (two breasts from one patient) were adjusted for. These results are presented as estimated means, adjusted differences and 95% confidence intervals (CI). Categorical variables are presented as absolute count (proportion) and were compared using X^2^ test or Fisher's exact test. A p-value <0.05 was considered statistically significant. All analyses were performed using IBM SPSS Statistics 28.0 (IBM Corp., Armonk, NY).

## 3 Results

Between July 2019 and February 2022, 199 patients undergoing a DIEP flap breast reconstruction were assessed for eligibility; 118 were included in the RCT (for the reasons of exclusion we refer to Fig. A2). In this interim analysis, 41 patients contributing 67 breasts were analyzed. The innervated group consisted of 19 patients contributing 29 breasts; the non-innervated group consisted of 22 patients contributing 38 breasts. In six DIEP-flaps, sensory nerve coaptation failed; three due to insufficient length, one due to a re-exploration in which the nerve coaptation was sacrificed, and in two cases no recipient nerve was identified. These numbers reflect the situation at the time of this interim analysis and may change until the RCT is finished.

Despite randomization, patients in the non-innervated group were on average older (50.2 ± 11.1 vs. 45.7 ± 8.6) and had a lower BMI (27.2 ± 3.3 vs. 28.1 ± 3.3) than patients in the innervated group. Non-innervated breasts were more often irradiated (47.4% vs. 20.7%). In the innervated group, more mastectomies were prophylactic and bilateral. Immediate reconstructions and previous implant reconstructions occurred more often in the innervated group. Mastectomy weight was higher in the innervated group (774.0 g vs. 618.6 g). Flap weight and ischemic time did not differ. All baseline characteristics are presented in Table 1.

**Table 1:** Patient characteristics.

	Innervated	Non-innervated
Total no. of patients	19	22
Total no. of DIEP flaps	29	38
Mean age ± SD, yr	45.7 ± 8.6	50.2 ± 11.1
Mean BMI ± SD, kg/m^2^	28.1 ± 3.3	27.2 ± 3.3
Hypertension	1 (5.3)	2 (9.1)
Previous lumpectomy^a^	3 (10.3)	5 (13.2)
Previous implant^a^	13 (44.8)	11 (28.9)
Reconstruction laterality		
Unilateral	5 (33.3)	10 (45.5)
Bilateral	14 (73.7)	12 (54.5)
Reconstruction timing^a^		
Immediate	12 (41.4)	13 (34.2)
Delayed	17 (58.6)	25 (65.8)
Mastectomy reason^a^		
Oncological	13 (44.8)	23 (60.5)
Prophylactic	16 (55.2)	15 (39.5)
Mean mastectomy weight ± SD, g^a^	774.0 ± 484.3	618.6 ± 338.3
Mean flap weight ± SD, g^a^	738.0 ± 348.7	714.3 ± 273.0
Mean ischemic time ± SD, min^a^	41.8 ± 18.7	40.9 ± 14.0
Oncologic treatment		
Radiation therapy^a^	6 (20.7)	18 (47.4)
Adjuvant chemotherapy	7 (36.8)	7 (31.8)
Neoadjuvant chemotherapy	6 (31.6)	9 (40.9)
Endocrine therapy	8 (42.1)	9 (40.9)
Immunotherapy	2 (10.5)	2 (9.1)

BMI, body mass index.

a breast is unit of analysis.

There were no significant differences in complication rates between the groups when comparing either according to *as treated* or *intention to treat* (Table A1, Table A2)*.* One major complication requiring re-intervention occurred; this was in the non-innervated group and concerned resolving a hemorrhage. Neuroma formation and neuropathic pain did not occur.

### 3.1 Sensory recovery

Preoperatively, sensibility was not significantly different between the groups (Table A3).

**Fig. 1:**
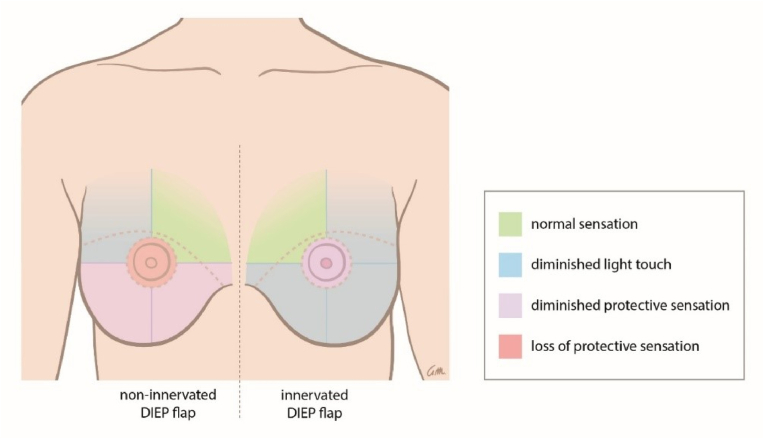
Schematic representation of the degree of sensory loss in non-innervated and innervated DIEP flaps.

At 24-months postoperatively, tactile thresholds measured with SWM were lower in the innervated flaps compared with non-innervated flaps in eight out of nine areas; statistically significant in areas 5–9. Protective sensation was diminished or lost in a larger surface area of the non-innervated breasts compared with the innervated breasts (Fig. 1). The mean SWM values of the flap skin and the total breast were also lower in innervated compared with non-innervated DIEP flaps (Table 2).

**Table 2:** Mean Semmes-Weinstein monofilament values per area at 24 months follow-up.

	*Innervated*	*Non-innervated*		
Area	Est. mean	Est. mean	Difference^a^ (95% CI)	*p*
**1**	3.07	2.91	−0.16 (-0.86, 0.53)	0.638
**2**	3.36	3.88	0.52 (-0.19, 1.23)	0.147
**3**	3.67	4.26	0.59 (-0.01, 1.18)	0.054
**4**	3.26	3.31	0.04 (-0.55, 0.63)	0.891
**5**	4.02	5.15	1.14 (0.54, 1.73)	<.001
**6**	4.30	5.31	1.00 (0.36, 1.65)	0.003
**7**	4.42	5.24	0.83 (0.29, 1.36)	0.003
**8**	4.45	5.12	0.67 (0.14, 1.20)	0.014
**9**	4.68	5.40	0.72 (0.25, 1.19)	0.003
**Mean native (1**–**4)**^b^	3.31	3.51	0.21 (-0.37, 0.78)	0.478
**Mean flap (5**–**9)**	4.48	5.20	0.72 (0.26, 1.18)	0.003
**Mean total**	3.90	4.54	0.65 (0.26, 1.03)	0.001

a Adjusted for patient ID (multilevel model).

b in large skin paddles areas 2 and 3 are located on the flap and only 1 and 4 are native skin.

**Fig. 2:**
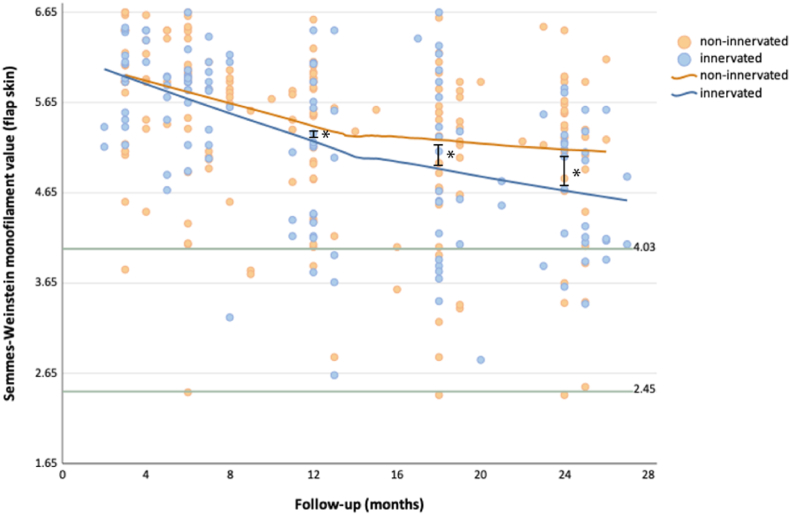
Scatter plot of the mean SWM value of the flap skin over time. The green lines indicate mean preoperative SWM values for immediate (2.45) and delayed (4.03) reconstructions. * = *p* < 0.05.

Sensibility was significantly better in the innervated DIEP flaps at 12 and 18 months as well (Table A4, Table A5). The difference between innervated and non-innervated flaps increased over time. Sensibility in the innervated flaps seemed to continue to improve after 24 months, whereas that in the non-innervated flaps seemed to stabilize (Fig. 2).

Static and dynamic touch thresholds measured with PSSD at 24 months postoperatively were more often imperceptible at the center of the non-innervated flaps (42.1% vs. 14.3%, p = 0.015; 21.1% vs. 3.6%, p = 0.041) (Table 3). Tactile thresholds measured with the PSSD were lower in innervated flaps. The difference was most profound in the center of the flap (47.8 ± 34.2 vs. 68.7 ± 32.8 g/mm^2^, p = 0.036; 22.3 ± 23.3 vs. 45.8 ± 36.7 g/mm^2^, p < 0.001) (Table 4).

**Table 3:** Proportion of DIEP flaps with no perceivable sensation by PSSD at 24-months follow-up.

		*Innervated*		*Non-innervated*		
Area		N (%) not felt		N (%) not felt		*p*
Medial upper quadrant	1-PS	1	(2.6)	0	(0)	1.000
	1-PM	0	(0)	0	(0)	NA
Lateral lower quadrant	1-PS	3	(10.7)	4	(10.5)	1.000
	1-PM	1	(3.6)	2	(5.3)	1.000
Flap center	1-PS	4	(14.3)	16	(42.1)	0.015
	1-PM	1	(3.6)	8	(21.1)	0.041

1-PS = 1-point static, 1-PM = 1-point moving.

**Table 4:** Mean PSSD value per area at 24 months follow-up.

		*Innervated*	*Non-innervated*		
Area		Est. mean	Est. mean	Difference (95% CI)^a^	*p*
Medial upper quadrant	1-PS	21.8	25.5	3.7 (-10.7, 18.2)	0.604
	1-PM	9.3	8.6	−0.7 (-5.8, 4.5)	0.794
Lateral lower quadrant	1-PS	32.3	50.7	18.4 (2.0, 34.9)	0.028
	1-PM	18.5	33.7	15.2 (0.4, 29.9)	0.044
Flap center	1-PS	47.8	71.2	23.4 (1.6, 45.1)	0.036
	1-PM	16.2	53.0	36.8 (19.2, 54.4)	<.001

1-PS = 1-point static, 1-PM = 1-point moving.

a Adjusted for patient ID (multilevel model).

Temperature thresholds were more often imperceptible in the lateral lower quadrant of the breast and center of the flap in non-innervated compared with innervated DIEP flaps (Table 5). This was most evident for heat pain thresholds (42.1% vs. 10.3%, p = 0.004, 57.9% vs. 34.5%, p = 0.057). No clear pattern was observed when comparing temperature threshold between the groups (Table A6).

**Table 5:** Proportion of DIEP flaps with no perceivable thermal sensation.

		*Innervated*		*Non-innervated*		
Area		N (%) not felt		N (%) not felt		*p*
Medial upper quadrant	WDT	1	(3.4)	2	(5.3)	1.000
	HPT	2	(6.9)	0	(0)	0.184
	CDT	2	(6.9)	1	(2.6)	0.574
	CPT	6	(20.7)	3	(7.9)	0.160
Lateral lower quadrant	WDT	9	(31.0)	16	(42.1)	0.353
	HPT	3	(10.3)	16	(42.1)	0.004
	CDT	5	(17.2)	13	(34.2)	0.121
	CPT	11	(37.9)	19	(50.0)	0.325
Flap center	WDT	16	(55.2)	20	(52.6)	0.836
	HPT	10	(34.5)	22	(57.9)	0.057
	CDT	12	(41.4)	20	(52.6)	0.361
	CPT	20	(69.0)	24	(63.2)	0.620

WDT = warm detection threshold, HPT = heat pain threshold, CDT = cold detection threshold, CPT = cold pain threshold.

## 4 Discussion

This interim analysis provides preliminary results of the first double-blind RCT that compares sensory recovery in innervated versus non-innervated DIEP flaps. The findings demonstrate superior sensibility and recovery of protective sensation in innervated DIEP flaps.

Sensory nerve coaptation is not a novelty in the field of autologous breast reconstruction. The first mention of an innervated flap for breast reconstruction was in 1992 by Slezak et al. [19] In 2013, Spiegel et al. proposed a refined technique: using the cutaneous branch of the third anterior intercostal nerve eliminated the need for an additional microsurgical field [14]. Several studies established that sensibility improves and returns sooner in innervated DIEP flaps, without compromising donor site sensibility [10,20,21]. This improves patient satisfaction and quality of life, marking the clinical relevance of the technique [22].

Despite these positive results, sensory nerve coaptation is still not widely adopted in clinical practice. The lack of double-blind randomized studies limits clinical implementation. Randomization and blinding reduce selection, performance and confirmation bias. A double-blind RCT therefore provides evidence with a yet unmet level of reliability.

Our results show lower tactile thresholds in innervated flaps. Importantly, the areas in the center of the DIEP flap (5–9), which are always located on the skin paddle of the flap, showed improved sensibility after sensory nerve coaptation. Hence, sensibility of the flap skin significantly improves by sensory nerve coaptation, regardless of reconstruction timing.

Peripherally, in the breast quadrants, sensibility seemed better in the innervated flaps, but the difference is smaller and not significant. The upper quadrants are also innervated by supraclavicular nerve branches that remain unharmed during mastectomy, which may partly preserve sensibility in these areas. The lower quadrants are more distant from the coapted nerve and may therefore regain less sensibility.

Note that areas 2 and 3 (lower medial and lateral quadrant) represent native skin in immediate reconstructions, and flap skin in delayed reconstructions. Subgroup analysis of immediate and delayed reconstructions would be valuable, but the sample size of this interim analysis is insufficient for reliable subgroup analysis. Hence, this remains reserved for the analysis of the completed RCT. We did calculate mean SWM values for flap skin and native skin per patient. This was determined individually and therefore accurately reflects the mean sensibility of flap and native skin, eliminating differences in flap size or shape related to immediate vs. delayed timing.

While in accordance with our previous work, it remains interesting that sensibility of the native skin also seems to improve by sensory nerve coaptation [20]. Underneath the native skin, the de-epithelialized flap is buried. Others suggest to remove both epidermis and dermis for better sensory recovery of the native skin, but there is no evidence suggesting superiority of either approach [23]. We hypothesize that sensibility of the native skin recovers partly by regeneration of nerves from adjacent skin, and partly from sprouting nerve fibers growing into the native skin from the underlying flap [24]. The latter, we believe, is mediated by sensory nerve coaptation. However, fundamental research is necessary to confirm these hypotheses and elucidate the mechanisms of reinnervation of both native and flap skin.

Sensibility of the native skin likely also improves if a nerve sparing mastectomy is performed [25]. In our hospital the supraclavicular nerve branches are spared, if possible, but sensory nerves passing through the breast tissue are not spared. This is a relevant and interesting topic for future research, to further improve postoperative sensibility.

Important for interpretation of our results is that the SWM data was analyzed using the unconverted SWM index values. This was preferred for their favorable statistical properties over the values converted to g/mm^2^, which would have violated the assumptions for our regression models. SWM index values represent a range of values and are therefore less precise than PSSD measurements, which presumably more adequately reflect the actual effect sizes.

It should be noted that there were differences in our baseline characteristics, despite randomization. This might be because this interim analysis does not include all randomized patients. Alternatively, this may be because randomization was conducted per patient, whereas some baseline characteristics are presented per breast (radiation therapy, mastectomy reason), and can therefore not be fully controlled by randomization at patient-level. For radiation therapy, there is some existing evidence that it negatively affects sensibility outcomes. However, previous work from our group did not show a significant effect of radiation therapy on postoperative sensibility; this may be because as much irradiated skin as possible is excised during a delayed reconstruction [10].

The clinical value of our quantitative results becomes clear when comparing protective sensation in both groups. The SWM and PSSD measurements demonstrated *loss of protective sensation* in all areas that correspond to the skin paddle of the flap in non-innervated flaps, whereas in the innervated flaps this improved to *diminished protective sensation*. According to Bell-Krotoski et al., *diminished protective sensation* means function is impaired, but the ability to respond to potential harmful stimuli remains. In *loss of protective sensation* there is risk to injuries [26]. Therefore, the shift from *loss* to *diminished* protective sensation in innervated flaps is highly relevant. Moreover, the lower quadrants of the non-innervated breasts had *diminished protective sensation*, which improved to *diminished light touch* in innervated breasts. Another clinically relevant improvement, since *diminished light touch* implies that the function remains within the normal range. Altogether, sensory nerve coaptation contributes significantly to the return of protective sensation in the reconstructed breast.

In thermal sensibility, our results varied. Thermal thresholds seemed more often imperceptible in non-innervated flaps. However, this was only significant for heat pain. This decreased ability to detect heat pain clinically translates to loss of protective sensation in non-innervated flaps. Potentially, the different nerve fibers that mediate tactile versus thermal and noxious stimuli (Aδ verus C-fibers) may regenerate differently [27]. Future research is needed to test this hypothesis and to better understand nerve regeneration.

Altogether, the described results are concordant with previous research, and thereby reinforce the evidence in favor of sensory nerve coaptation. No associations between sensory nerve coaptation and complications (specifically neuroma, neuropathic pain) were found.

Although this interim analysis presents important quantitative data, patient-reported outcomes like the BREAST-Q are essential to determine the clinical value of sensory nerve coaptation [28]. This will be included in the analysis of the completed RCT. This interim analysis comprises 41 of the 118 patients from the full trial. The patients were not selected by any means other than completion of the 2-year follow-up by June 2022. The results of the completed RCT remain greatly important, as the larger sample size enables subtle differences to also reach statistical significance. The completed RCT will also have sufficient sample size to include subgroup analysis for immediate and delayed reconstructions. In addition, it enables reliable assessment of the quality-of-life outcomes, based on which the sample size was calculated.

An arguable limitation of this study, is that it was analyzed and presented according to the *as treated* principle. This affects the randomization and may induce selection bias, as six breasts allocated to the innervated group crossed-over to the non-innervated group. However, the *as treated* principle suited the purpose of this interim analysis best: to provide estimates of the efficacy and feasibility of sensory nerve coaptation, in a double-blind setting. The purpose of the full RCT is distinctly different: to steer future application and clinical implementation. That requires thorough investigation of the effectiveness of the intervention, taking realistic failure rates into account. Ultimately, this interim analysis and the full RCT serve different purposes, requiring different analysis.

Finally, sensation is a complex and multifaceted concept, that is not fully reflected merely by tactile and thermal thresholds. Therefore, we encourage other quantitative and qualitative measurements of sensation to be explored. Besides this, the reliability and repeatability of sensory testing is often debated [29,30]. Hence, the complexity of sensibility and its assessment still pose challenges in current research on innervated breast reconstruction.

## 5 Conclusion

This interim analysis of our double-blind randomized controlled trial indicates that sensibility in innervated DIEP flaps recovers better than in non-innervated DIEP flaps. Therefore, we encourage the continuation of clinical research into this valuable technique and support clinical implementation. Additional research into the surgical technique, as well as fundamental research on nerve regeneration will improve understanding of the different facets of sensation, and may enable further improvement of sensory recovery in reconstructed breasts. Although our results are encouraging, its preliminary nature requires the final results of the complete RCT to be awaited for additional and definite conclusions.

## CRediT authorship contribution statement

**Jeske M. Bubberman:** Writing – review & editing, Writing – original draft, Project administration, Methodology, Investigation, Formal analysis, Data curation, Conceptualization. **Lloyd Brandts:** Writing – review & editing, Visualization, Methodology, Formal analysis, Data curation. **Sander M.J. van Kuijk:** Writing – review & editing, Visualization, Methodology, Formal analysis, Data curation, Conceptualization. **René R.W.J. van der Hulst:** Writing – review & editing, Supervision, Resources, Methodology, Investigation, Funding acquisition, Conceptualization. **Stefania M.H. Tuinder:** Writing – review & editing, Supervision, Resources, Methodology, Investigation, Funding acquisition, Conceptualization.
