# Supplementary material for: The efficacy of sensory nerve coaptation in DIEP flap breast reconstruction – Preliminary results of a double-blind randomized controlled trial
Source: Breast. 2024 Feb 9;74:103691. doi: 10.1016/j.breast.2024.103691 (PMC10904190; doi:10.1016/j.breast.2024.103691)
Supplement: Multimedia component 1 [file mmc1.pdf]

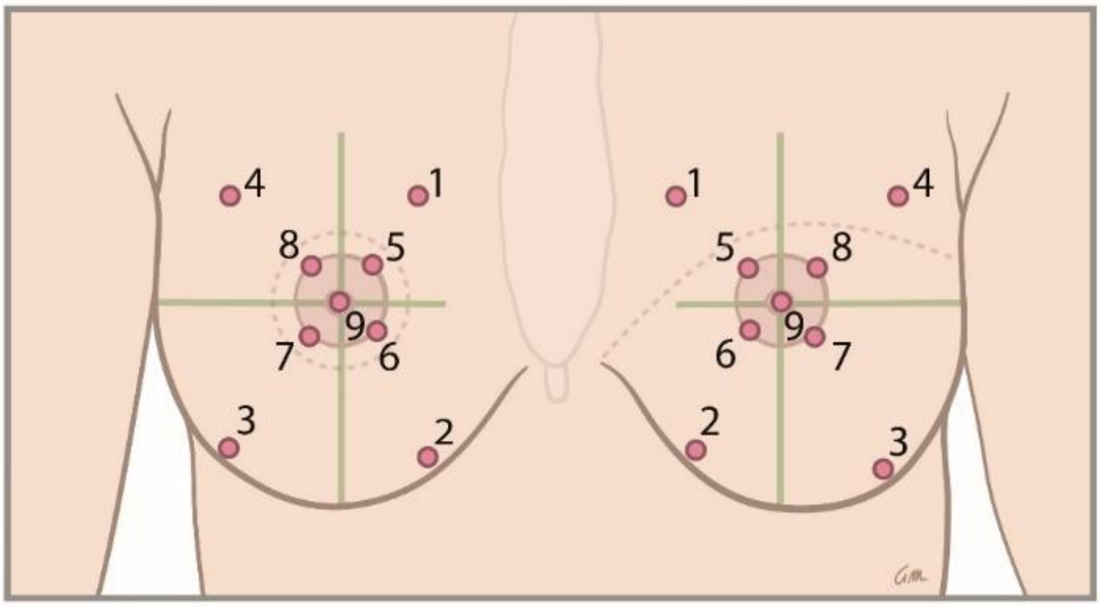

Figure A1. Schematic overview of the nine sensory testing areas per breast in immediate (right breast) and delayed (left breast) DIEP flap breast reconstruction.

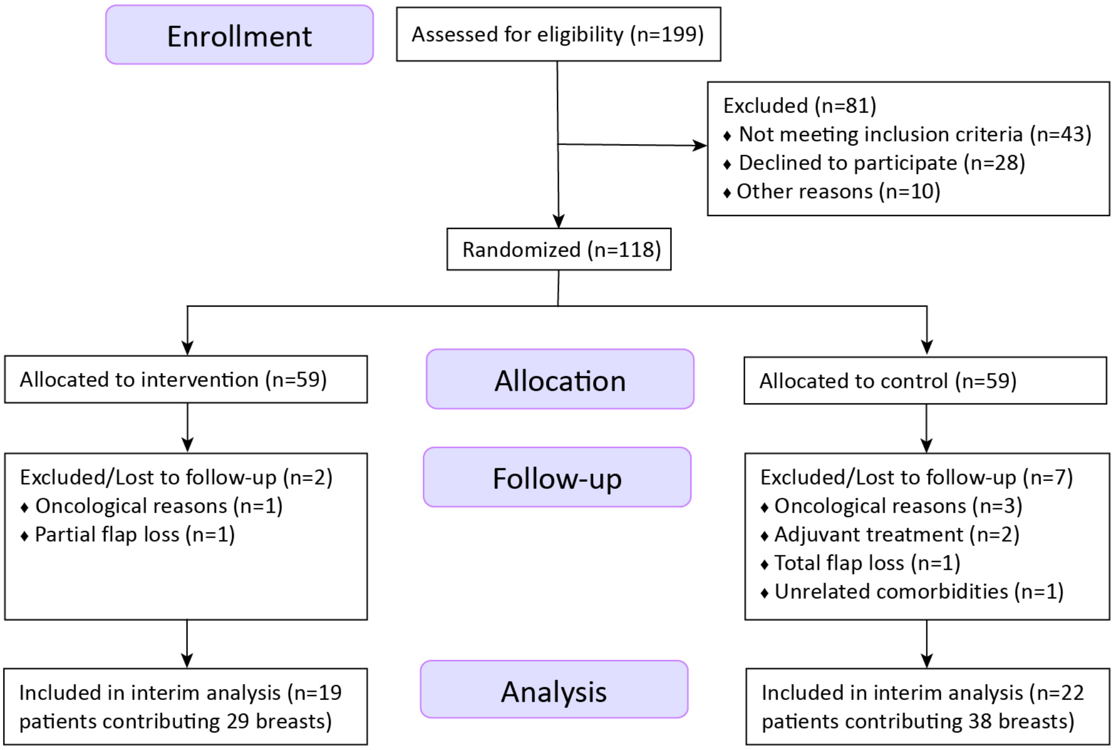

Figure A2. CONSORT flow chart of patient inclusion of the complete randomized controlled trial.

Table A1. Complication rates analyzed according to *as treated*

	Innervated	Non-innervated	*p*
Intraoperative complications	4 (13.8)	9 (23.7)	0.365
Venous revision	0 (0)	5 (13.2)	0.064
Arterial revision	4 (13.8)	3 (7.9)	0.690
Other	0 (0)	1 (2.6)	1.000
Minor complications (breast)	5 (17.2)	14 (36.8)	0.078
Fat necrosis	2 (6.9)	7 (18.4)	0.280
Infection	1 (3.4)	1 (2.6)	1.000
Impaired wound healing	1 (3.4)	8 (21.1)	0.067
Hematoma	0 (0)	1 (2.6)	1.000
Other	2 (6.9)	1 (2.6)	0.574
Breast re-explorations	0	1 (2.6)	1.000
Donor site complications*	5 (26.3)	3 (13.6)	0.436
Seroma	2 (10.5)	0 (0)	0.209
Impaired wound healing	3 (15.8)	3 (13.6)	1.000
Infection	1 (5.3)	0 (0)	0.463
Bulging	1 (5.3)	0 (0)	0.463

*patient as unit of analysis

Table A2. Complication rates analyzed according to *intention to treat*

	Innervated	Non-innervated	*p*
Intraoperative complications	7 (20.0)	6 (18.8)	0.897
Venous revision	1 (2.9)	4 (12.5)	0.185
Arterial revision	5 (14.3)	2 (6.3)	0.431
Other	1 (2.9)	0 (0)	1.000
Minor complications (breast)	9 (25.7)	10 (31.3)	0.616
Fat necrosis	4 (11.4)	5 (15.6)	0.727
Infection	2 (5.7)	0 (0)	0.493
Impaired wound healing	2 (5.7)	7 (21.9)	0.075
Hematoma	0 (0)	1 (3.1)	0.478
Other	3 (8.6)	0 (0)	0.240
Breast re-explorations	1 (2.9)	0 (0)	1.000
Donor site complications*	5 (23.8)	3 (15.0)	0.697
Seroma	2 (9.5)	0 (0)	0.488
Impaired wound healing	3 (14.3)	3 (15.0)	1.000
Infection	1 (4.8)	0 (0)	1.000
Bulging	1 (4.8)	0 (0)	1.000

*patient as unit of analysis

Table A3. Preoperative Semmes-Weinstein monofilament values

Area	*Innervated* Est. mean	*Non-innervated* Est. mean	Difference[a] (95% CI)	*p*
**1**	2.33	2.54	0.21 (-0.25, 0.67)	0.362
**2**	2.66	2.80	0.15 (-0.41, 0.70)	0.604
**3**	3.11	3.48	0.37 (-0.45, 1.19)	0.370
**4**	2.64	2.84	0.20 (-0.40, 0.79)	0.518
**5**	3.75	4.03	0.29 (-0.54, 1.12)	0.495
**6**	3.76	4.19	0.43 (-0.38, 1.24)	0.292
**7**	3.74	4.24	0.50 (-0.29, 1.27)	0.210
**8**	3.88	4.09	0.21 (-0.61, 1.03)	0.607
**9**	4.04	4.43	0.39 (-0.36, 1.15)	0.303
**Mean total**	3.55	3.97	0.42 (-0.35, 1.18)	0.283

[a] Adjusted for patient ID (multilevel model)

Table A4. Semmes-Weinstein monofilament values at 12 months follow-up

Area	*Innervated* Est. mean	*Non-innervated* Est. mean	Difference[a] (95% CI)	*p*
**1**	3.19	3.29	0.09 (-0.43, 0.62)	0.726
**2**	3.72	4.39	0.67 (0.06, 1.27)	0.031
**3**	4.19	4.69	0.49 (-0.10, 1.09)	0.102
**4**	3.02	3.49	0.48 (0.03, 0.93)	0.037
**5**	4.85	5.13	0.28 (-0.18, 0.74)	0.228
**6**	4.98	5.39	0.41 (0.03, 0.79)	0.036
**7**	4.93	5.36	0.43 (-0.00, 0.87)	0.052
**8**	4.81	5.28	0.47 (-0.01, 0.95)	0.057
**9**	4.92	5.47	0.55 (0.14, 0.95)	0.009
**Mean native (1-4)**	3.46	3.75	0.28 (-0.16, 0.73)	0.201
**Mean flap (5-9)***	4.86	5.38	0.52 (0.09, 0.95)	0.020
**Mean total**	4.30	4.73	0.43 (0.12, 0.75)	0.008

[a] Adjusted for patient ID (multilevel model)

*in large skin paddles areas 2 and 3 are located on the flap and only 1 and 4 are native skin.

Table A5. Semmes-Weinstein monofilament values at 18 months follow-up

Area	*Innervated* Est. mean	*Non-innervated* Est. mean	Difference[a] (95% CI)	*p*
**1**	3.03	3.02	-0.01 (-0.43, 0.40)	0.957
**2**	3.69	3.92	0.23 (-0.46, 0.92)	0.508
**3**	4.02	4.33	0.31 (-0.31, 0.93)	0.322
**4**	3.02	3.18	0.16 (-0.26, 0.58)	0.453
**5**	4.48	5.07	0.59 (-0.02, 1.20)	0.056
**6**	4.52	5.28	0.76 (0.25, 1.28)	0.004
**7**	4.58	5.32	0.74 (0.33, 1.15)	<0.001
**8**	4.52	5.12	0.59 (0.02, 1.17)	0.043
**9**	4.68	5.32	0.64 (0.11, 1.18)	0.020
**Mean native (1-4)**	3.26	3.53	0.26 (-0.15, 0.67)	0.208
**Mean flap (5-9)***	4.62	5.17	0.55 (0.10, 1.00)	0.017
**Mean total**	4.01	4.56	0.55 (0.19, 0.92)	0.003

[a] Adjusted for patient ID (multilevel model)

*in large skin paddles areas 2 and 3 are located on the flap and only 1 and 4 are native skin.

Table A6. Mean thermal thresholds per area of innervated and non-innervated DIEP flaps

Area		*Innervated* Est. mean	*Non-innervated* Est. mean	Difference[a] (95% CI)	*p*
**1**	**WDT**	41.0	39.9	-1.17 (-3.46, 1.12)	0.313
	**HPT**	45.8	44.7	-1.12 (-2.99, 0.75)	0.236
	**CDT**	22.2	25.0	2.81 (-0.74, 6.35)	0.119
	**CPT**	11.0	15.5	4.49 (-1.07, 10.05)	0.112
**3**	**WDT**	43.0	45.0	1.99 (-0.17, 4.14)	0.070
	**HPT**	46.7	47.8	1.11 (-0.10, 2.32)	0.072
	**CDT**	18.5	13.7	-4.78 (-10.76, 1.21)	0.116
	**CPT**	9.7	5.6	-4.16 (-8.92, 0.60)	0.086
**9**	**WDT**	46.4	45.8	-0.62 (-3.06, 1.81)	0.610
	**HPT**	47.3	49.0	1.67 (-0.03, 3.38)	0.054
	**CDT**	11.0	10.3	-0.74 (-5.46, 3.98)	0.754
	**CPT**	3.9	4.0	0.08 (-2.78, 2.95)	0.953

WDT=warm detection threshold, HPT=heat pain threshold, CDT=cold detection threshold, CPT=cold pain threshold

[a] Adjusted for patient ID (multilevel model)
